# Melanopic equivalent daylight illuminance of 2 lx maintains and restores physiological and neurophysiological circadian rhythms in rats

**DOI:** 10.1038/s41598-026-49695-6

**Published:** 2026-04-25

**Authors:** Seohyeon Kim, Hyeji Yu, Kang-Min Choi, Jonggyun Kim, Seulgi Kim, Yun Jae Eo, Keyong Nam Lee, Chang-Hwan Im, Young Rag Do, Seung Min Lee

**Affiliations:** 1 School of Electrical Engineering, Kookmin University, Seoul, 02707 South Korea; 2 Department of Chemistry, Kookmin University, Seoul, 02707 South Korea; 3 Department of Biomedical Engineering, Hanyang University, Seoul, 04763 South Korea; 4 Department of Electronic Engineering, Seoul National University of Science and Technology, 01811 Seoul, South Korea

**Keywords:** Neuroscience, Physiology

## Abstract

Understanding the non-visual effects of light is increasingly important, as circadian disruption from inappropriate exposure negatively impacts sleep, metabolism, and cognition. However, artificial lighting is rarely designed from a non-visual illuminance perspective, and its effects on biological rhythms remain incompletely evaluated. Here, we investigated three lighting conditions in rats: conventional light–dark (L/D), and two novel settings with matched visual illuminance but differing nighttime rat melanopic equivalent daylight illuminance (2–4 lx; rat mel EDI), enabled by tunable four-package white light-emitting diodes. Circadian rhythms were assessed using physiological (core body temperature, heart rate, locomotor activity) and neurophysiological markers (non-rapid eye movement sleep index, θ–γ cross-frequency coupling) across rhythmicity (periodicity, amplitude), stability (inter-daily stability, intra-daily variability), and state classification (Gaussian mixture modeling, k-means clustering). Relative to 4 lx, the 2 lx lighting most closely resembled L/D in minimizing rhythm loss and maintaining sleep–wake synchrony. Furthermore, after circadian disruption, switching to the 2 lx lighting restored physiological and neurophysiological markers. These findings indicate that spectrally optimized 2 lx lighting is a viable strategy to maintain or reinstate circadian health when nighttime illumination is unavoidable.

**Supplementary Information:**

The online version contains supplementary material available at 10.1038/s41598-026-49695-6.

## Introduction

To accommodate daily environmental changes and maintain homeostasis, mammals show circadian rhythms with a periodicity of approximately 24 h in behavior and physiology, such as in their sleep patterns and metabolism.^1,2^ Light is the dominant zeitgeber that entrains circadian phase to the external day–night cycle.^3–6^ Light information relevant to circadian entrainment is processed through integrated retinal pathways and relayed via melanopsin-expressing intrinsically photosensitive retinal ganglion cells (ipRGCs) to the suprachiasmatic nucleus, driving non-visual responses including circadian entrainment.^7–9^.

Light from various artificial lighting sources delivered through this non-visual pathway is known to disrupt the circadian rhythm.^10^ Unlike what is seen in nature, modern lifestyles expose people to altered lighting environments characterized by reduced daytime exposure to sufficiently bright light and increased evening light exposure from artificial sources,^11^ which causes disorders affecting sleep,^12^ metabolism,^13^ and mood.^14^ In particular, night shift workers, who experience a significant mismatch between their circadian clock and the external lighting environment, have increased rates of cancer, cardiovascular and metabolic disorders, as well as increased prevalence of behavioral health and mental disorders.^15–19^.

Since the non-visual effects of light significantly impact health, rodent studies have investigated how mis-timed, wavelength-dependent light exposure affects metabolism and disease while controlling other environmental variables. Exposure to light at night or other inappropriate times (even 5 lx of dim white light) is known to cause weight gain,^20,21^ depression-like reactions,^22,23^ and worse stroke outcomes^24^ are known to occur in both diurnal and nocturnal rodents.^23^ In addition, the influence of both light illuminance and wavelength has been found to be very significant because of the photosensitivity of melanopsin: the highest in blue light (~ 480 nm) and lowest in red light (> 600 nm).^25,26^ At the same illuminance level of 5 lx, dim blue light has been shown to impair emotional responses more than dim red light,^27^ whereas 10 lx red light has been reported not to affect sleep-awake behavior.^28^ However, many prior studies relied on monochromatic or simplified spectra, which can limit direct translation to real-world polychromatic lighting.

Recent studies have demonstrated that melanopsin-weighted light can be modulated through spectral tuning and short-wavelength filtering approaches, and that these manipulations can influence melatonin-related outcomes.^29–31^ To quantify melanopsin-weighted light under polychromatic conditions in this rodent study, we use rat melanopic equivalent daylight illuminance (rat mel EDI), a spectrum-aware metric referenced to standard daylight that enables comparison of melanopsin-weighted light levels across spectra.^32^ Nevertheless, systematic evaluation remains limited in the literature, particularly for long-term polychromatic nighttime lighting designs that match visual illuminance, parametrically separate rat mel EDI, and simultaneously quantify multimodal physiological and ECoG-derived neurophysiological endpoints.

In the present study, the appropriate spectrum and intensity of light that satisfies low non-visual illuminance with minimal impact on biological rhythms while enabling visualization and activity in the surrounding environment was explored. For this purpose, Sprague-Dawley (SD) rats were housed in individual cages in which the lighting condition was controlled using tunable four-package white light-emitting diodes (LEDs), which produce a tunable circadian effect for melatonin suppression/secretion.^33^ This tunable four-package white LEDs made it possible to design various lighting conditions with different non-visual illuminance despite having the same visual illuminance (VIL). In addition to the usual light (L) and dark (D), circadian illuminance high (CIL-H) and circadian illuminance low (CIL-L) were designed to imitate sunrise and sunset to increase or lower the non-visual illuminance compared to the VIL, respectively. Under these various lighting conditions, multiple biosignals such as core body temperature (CBT), heart rate (HR), locomotor activity (LA), and electrocorticogram (ECoG) were acquired over 10 days. Analysis from the circadian rhythm perspective was performed in two categories: physiological changes through CBT, HR, and LA monitoring and neurophysiological changes through non-rapid eye movement (NREM) and θ-γ cross-frequency coupling (CFC) extracted from ECoG. Subsequently, lighting conditions that ensured visual illumination while having minimal adverse effects on circadian rhythms were identified through analysis of the results using multiple methods. In addition, we investigated whether our approach negatively affected circadian rhythms or could realign them, in view of its potential for post-disruption circadian recovery. Our approach could provide insight into how to design light sources that provide healthier nighttime lighting from a circadian rhythm perspective in situations where exposure to inappropriate light sources is inevitable.

## Results

### The non-visual illuminance lighting conditions

To assess the effects of non-visual illuminance on circadian rhythms, the following three lighting conditions with 12 h/12 h cycles were used in the experiments: L/D and two spectrally engineered conditions in which visual illuminance was matched but nighttime rat mel EDI differed (2–4 lx; rat mel EDI), hereafter referred to as the 2 lx and 4 lx conditions, respectively. These engineered conditions consisted of CIL-H during the daytime phase and CIL-L during the nighttime phase. The VIL for CIL-H and L was the same (185 lx), whereas their rat mel EDI values were different (216 vs. 161 lx) (Table S1). The two CIL-L nighttime spectra had the same VIL (30 lx) but different nighttime rat mel EDI (2–4 lx) based on how much the blue wavelength region is enhanced or reduced under the same VIL conditions. The spectral irradiance for each lighting condition is shown in Fig. S1. The designed lighting conditions were applied to SD rats implanted with wireless telemetry devices in individual light-controllable cages (Fig. S2). All subjects were exposed to the L/D condition for at least 5 days as an entrainment period. Afterward, some of them were maintained under this condition to preserve their circadian rhythms (the control group) while the others were assigned to experimental groups and exposed to one of the other lighting conditions.

### Physiological circadian rhythm changes under the three lighting conditions

**Fig. 1:**
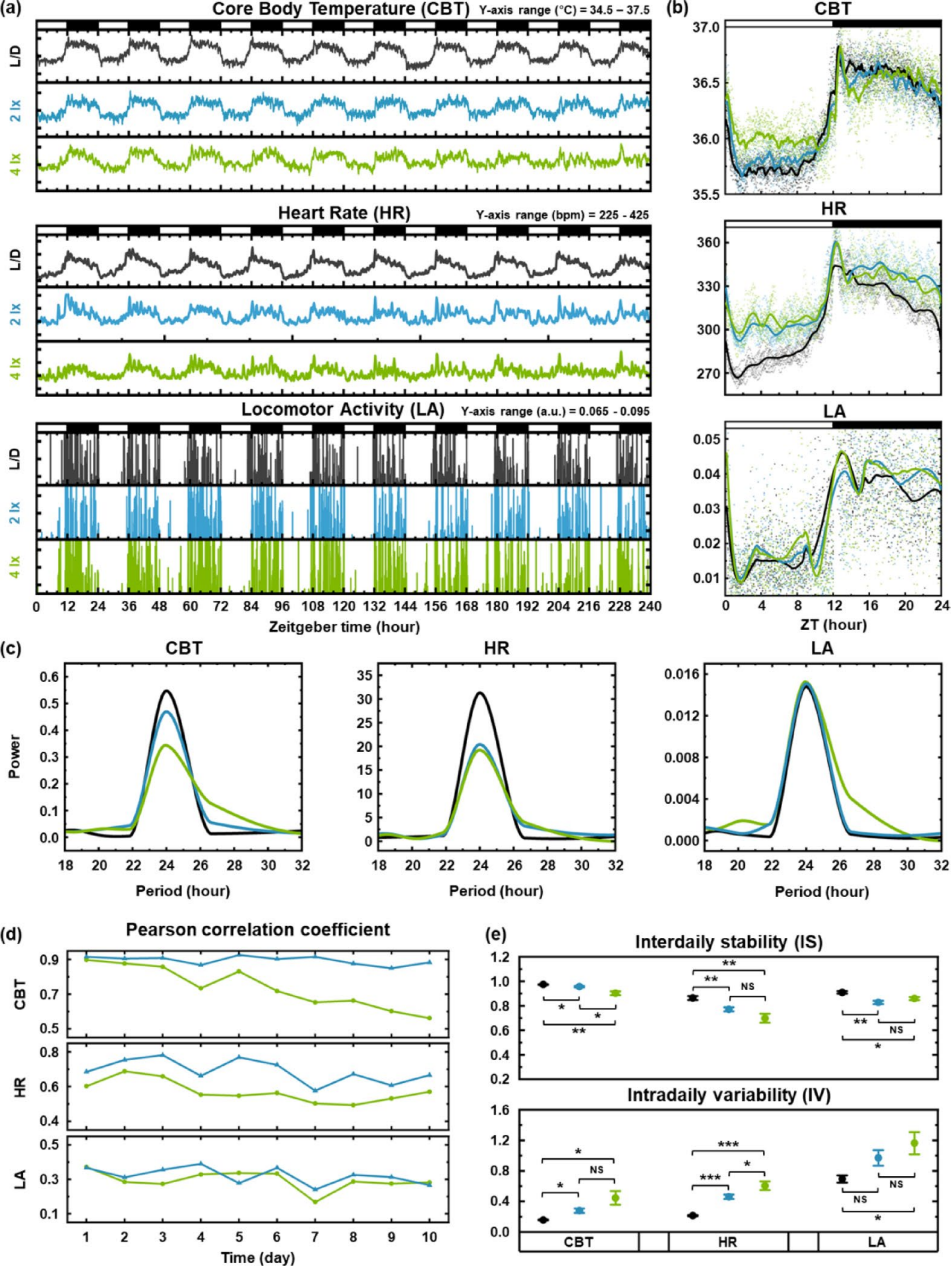
Physiological parameters and circadian rhythm metrics under various lighting conditions over 10 days following the entrainment period. **a** Time-series plots for core body temperature (CBT), heart rate (HR), and locomotor activity (LA). **b** Daily averaged profiles for CBT, HR, and LA. **c** Periodograms for CBT, HR, and LA calculated using fast-Fourier transform (FFT) with B-spline smoothing. **d** Pearson correlation coefficient (*r*) values for daily CBT, HR, and LA values relative to those for the L/D condition. **e** Inter-daily stability (IS) and intra-daily variability (IV) of CBT, HR, and LA. Black/white bars indicate nighttime/daytime, respectively. L/D, *n* = 11 (black); 2 lx, *n* = 9 (blue); 4 lx, *n* = 7 (green). Two-tailed Welch’s two-sample t-tests, with Holm–Bonferroni correction for multiple comparisons: *p < 0.05, **p < 0.01, ***p < 0.001.

After the L/D entrainment period, group-averaged physiological time-series data related to the circadian cycle (CBT, HR, and LA) under various lighting conditions were acquired for 10 days (Fig. 1a). For the L/D group, CBT, HR, and LA all increased during nighttime (zeitgeber time; ZT 12–24) and decreased during daytime (ZT 0–12), which is as expected since the conditions were the same as during entrainment. Moreover, although the 2 and 4 lx light groups had the same daytime but different nighttime lighting conditions, the biosignals for these groups were markedly different. It is noteworthy that the 2 lx condition relatively well maintained the periodic amplitudes and circadian rhythms of the biosignals similar to those for the L/D condition, whereas the 4 lx condition decreased the amplitudes of the biosignals over time, thereby indicating disturbance of the circadian rhythms.

To clearly visualize the biosignal trends, daily averaged profiles of CBT, HR, and LA were obtained by averaging the 10-day recordings (Fig. 1b). Relative to the L/D condition, the daytime–nighttime differences, quantified as peak-to-peak values, decreased by 4.7% and 19% for CBT under the 2 lx and 4 lx conditions, respectively, and by 11% and 19% for HR. For LA, the daytime–nighttime difference decreased by 4.6% under the 2 lx condition but increased by 1.6% under the 4 lx condition. Overall, the daytime–nighttime differences tended to decrease from L/D to 2 lx to 4 lx, while LA showed minimal changes under both 2 lx and 4 lx.

The periodicities of the biosignals for each lighting condition were compared and analyzed in the frequency domain using periodograms and fast-Fourier transform (FFT), after which B-spline smoothing was applied to identify trends in the FFT signals. As can be seen in Fig. 1c, under the L/D condition, the periodogram for the biosignals shows a peak value at 24.0 h. For the 2 lx and 4 lx conditions compared to the L/D condition, the CBT peak values decreased by 13% and 32% with periods of 24.0 and 24.3 h, respectively; the HR peak values decreased by 32% and 35%, both with periods of 24.0 h; and the LA peak values decreased by 0.7% and 2.6%, both with periods of 24.0 h. The full width at half maximum (FWHM) values for the L/D, 2 lx, and 4 lx conditions were 3.11, 3.50, and 4.12 for CBT; 3.21, 3.54, and 3.69 for HR; and 2.52, 2.69, and 3.17 for LA: a narrower FWHM indicates a sharper and more stable circadian rhythm whereas a broader FWHM reflects a more dispersed and variable one.

These disturbances in the circadian rhythms caused decreased amplitudes and phase delays, which was quantified by Pearson’s correlation coefficient (*r*) between each day’s CBT, HR, and LA profiles derived from the 10-day recordings in Fig. 1a and the corresponding profiles under the L/D condition (Fig. 1d). Similar to the trend in the periodicity analysis, the Pearson’s *r* values for CBT gradually decreased over time in the order of 2 lx and 4 lx, with the final values being 0.850 and 0.562, respectively. Although the differences between the HR and LA for the 2 and 4 lx conditions were relatively small compared to those for CBT, both of them showed a similar trend (0.576 and 0.493 for HR, 0.240 and 0.168 for LA, respectively), with the 2 lx condition attaining slightly higher values than the 4 lx condition. In addition to confirming the change in circadian rhythm over time under each lighting condition, inter-daily stability (IS) and intra-daily variability (IV) were adopted for quantitative analysis from the circadian rhythm perspective of (Fig. 1e); these criteria indicate how stable the circadian rhythm is over multiple days and indicates the rate of shifting between rest and activity by reflecting the fragmentation of the rhythm, respectively.^34^ As expected based on the previous results, the IS values for CBT and HR gradually decreased in the order of the L/D, 2 lx, and 4 lx conditions (0.973, 0.956, and 0.902 for CBT; 0.864, 0.771, and 0.698 for HR, respectively). Moreover, the values for LA were 0.909, 0.827, and 0.859, respectively; i.e., slightly lower for 2 lx than for 4 lx. (Welch one-way ANOVA; CBT, *p* = 1.08 × 10^−3^, η^2^ = 0.599; HR, *p* = 9.25 × 10^−4^, η^2^ = 0.512; LA, *p* = 7.71 × 10^−4^, η^2^ = 0.533) Meanwhile, the CBT, HR, and LA values for IV also gradually increased in the same order of L/D, 2 lx, and 4 lx (0.158, 0.279, and 0.446; 0.215, 0.461, and 0.605; and 0.694, 0.970, and 1.16). In particular, decreasing IS and increasing IV were in the order of L/D, 2 lx, and 4 lx, which means that periodicity was stably maintained from the circadian rhythm perspective. (Welch one-way ANOVA; CBT, *p* = 8.40 × 10^−4^, η^2^ = 0.340; HR, *p* = 5.51 × 10^−7^, η^2^ = 0.682; LA, *p* = 8.48 × 10^−3^, η^2^ = 0.270)

From a comprehensive physiological perspective, the circadian rhythms were disturbed under the lighting conditions other than L/D. There were also differences in the way each biosignal was disturbed according to the lighting conditions: the peak amplitude for HR changed prominently, the peak period for LA changed, and both the peak amplitude and period changed for CBT. However, for the 4 lx and 2 lx conditions, the peak periods for CBT, HR, and LA were maintained, indicating that no marked change in periodicity was detected by the periodogram analysis. In particular, the 2 lx condition maintained the circadian rhythm significantly better than 4 lx condition to a degree comparable to the L/D condition, as supported by the higher power of the periodogram, less decrease in the Pearson’s *r* values, as well as higher biosignal values for IS and lower ones for IV.

### Circadian rhythm changes in sleep-awake and cognition-associated neural metrics under the lighting conditions

**Fig. 2:**
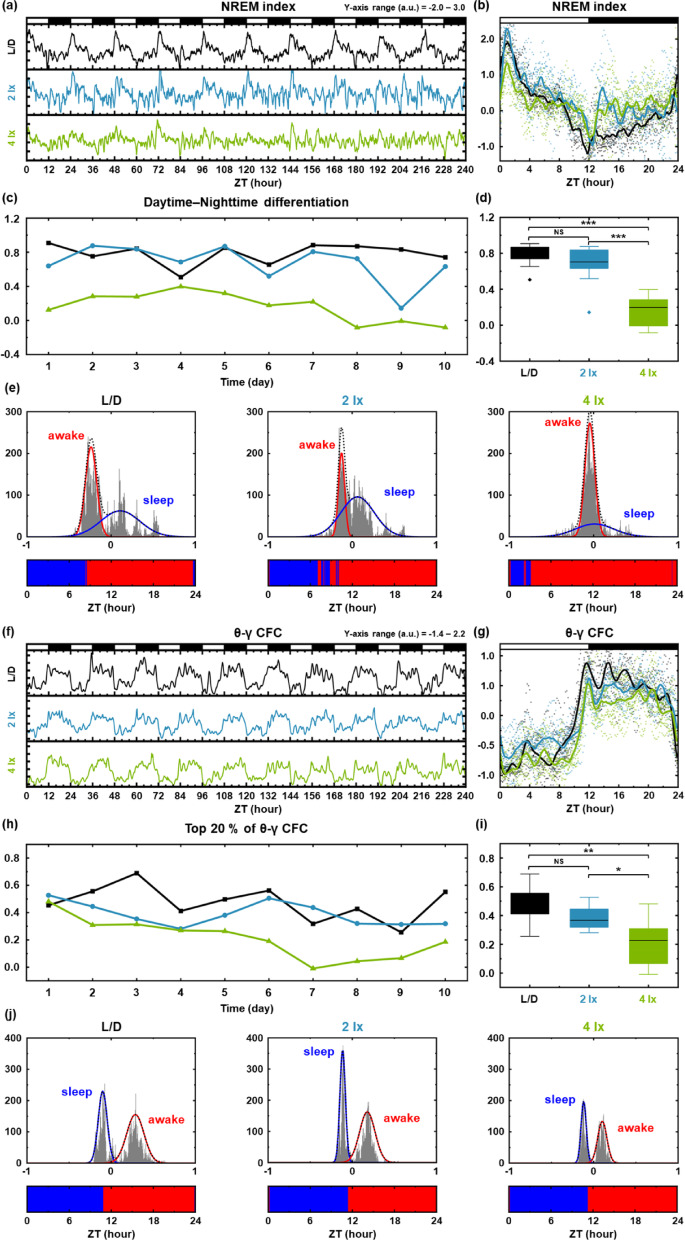
Neurophysiological parameters and circadian rhythm metrics under the four lighting conditions. **a** Time-series plots of non-rapid eye movement (NREM) index over 10 days following the entrainment period. **b** Daily averaged NREM index values. **c** Daytime–nighttime differences in NREM index values across 10 days. **d** A box plot of daytime–nighttime differences in the NREM index across 10 days. **e** (Top) Gaussian mixture model (GMM) distribution of NREM index values with bimodal Gaussian fitting to classify the sleep (blue) and awake (red) states. (Bottom) *k*-means clustering analysis of NREM index values with two predefined clusters for the sleep and awake states. **f** Time-series plots of θ-γ cross-frequency coupling (CFC) over 10 days following the entrainment period. **g** Daily averaged θ-γ CFC values. **h** Top 20% θ-γ CFC values across 10 days. **i** A box plot of the top 20% θ-γ CFC values across 10 days. **j** (Top) GMM distribution of θ-γ CFC values with bimodal Gaussian fitting to classify sleep (blue) and awake (red) states. (Bottom) *k*-means clustering results for the θ-γ CFC values with two predefined clusters for sleep and awake. Black/white bars indicate nighttime/daytime, respectively. L/D, *n* = 11 (black); 2 lx, *n* = 9 (blue); 4 lx, *n* = 7 (green). Two-tailed Welch’s two-sample t-tests, with Holm–Bonferroni correction for multiple comparisons: *p < 0.05, **p < 0.01, ***p < 0.001.

To analyze the effects of the various lighting conditions on neurophysiological changes, the NREM index was used for sleep analysis, and θ-γ CFC was quantified as a cognition-associated neural metric (Fig. 2). The NREM index was extracted from the ECoG output over 10 days under various lighting conditions following the entrainment period: high values were obtained during daytime when the nocturnal rodents were sleeping and low values during nighttime when they were awake (Fig. 2a). Similar to the physiological data, these periodic amplitudes were largest under the L/D condition and smallest under the 4 lx condition, with the 2 lx condition being in between. The peak values for the daily average NREM index were observed during ZT 0–2 (immediately after falling asleep): similar values for the L/D and 2 lx conditions, and lower values for the 4 lx condition (Fig. 2b). In addition, in terms of NREM index for the whole nighttime, the lowest value was obtained for the L/D condition, followed by similar values for the 2 and 4 lx conditions. Therefore, when comparing the peak-to-peak NREM index values with those for the L/D condition, the 2 lx and 4 lx conditions showed decreases of 1.7% and 20%, respectively.

To assess the impact of the lighting conditions on sleep-awake differentiation over time, differences between the NREM index values for daytime and nighttime for each of them were calculated (Fig. 2c). Immediately after entrainment and for the L/D condition, the sleep and awake times clearly divided into nighttime and daytime, respectively, ensured that the differences in NREM index values ​​were relatively high. In comparison, the minimum values ​​for L/D, 2 lx, and 4 lx conditions were 0.506, 0.144, and − 0.084, respectively. To statistically assess the temporal robustness of sleep-awake differentiation for each lighting condition, we analyzed the distribution of the daytime–nighttime NREM index differences across the 10-day recording period using a box plot (Fig. 2d). The L/D condition showed the largest median difference and a narrow interquartile range, thereby indicating well-preserved circadian organization over time. The 2 lx condition exhibited a slightly lower median but maintained a relatively consistent day-to-day pattern during the 10-day exposure period, suggesting that circadian rhythmicity was largely retained. In contrast, the 4 lx condition showed much lower median differences and increased variability, exhibiting several days with NREM index difference values near or below zero. These patterns imply a progressive breakdown of day–night differentiation in sleep-related neurophysiological changes with elevated light intensity at night. Interestingly, no significant difference was found between the L/D and 2 lx conditions, while both were significantly different from the 4 lx condition. This implies that sleep rhythmicity under the 2 lx condition was similar to L/D but functionally distinct from the 4 lx condition, which implies that the 2 lx condition preserves circadian regulation to a meaningful extent (Welch one-way ANOVA; *p* = 2.30 × 10^− 7^, η^2^ = 0.725).

The actual sleep and awake times may not match time divisions into day and night, so it is important to clearly analyze the difference between the NREM index values at the sleep and awake times. Therefore, using the 24-hour daily-averaged NREM index profile (Fig. 2b), the NREM index values ​​were divided into two clusters for sleep and awake through Gaussian mixture model (GMM) analysis with bimodal Gaussian fitting. As can be seen in Fig. 2e, the intervals between these peaks for the L/D, 2 lx, and 4 lx conditions were 0.35, 0.19, and − 0.06, respectively. Moreover, the NREM index values for the awake and sleep states for the 2 lx and L/D conditions were clearly distinguishable, whereas the 4 lx condition had a greatly decreased interval between the peaks, making it difficult to distinguish between the awake and sleeping states. Sleep-awake grouping was also performed using *k-*means clustering, which is a data-driven method. In both the L/D and 2 lx conditions, sleep states were synchronized with daytime at ZT 0–8, with awake/sleep proportions of 63.3%/36.7%, and 65.2%/34.8%, respectively. On the other hand, for the 4 lx condition, where sleep and awake classification was difficult to separate via GMM analysis, the awake/sleep proportion was 88.8%/11.2%. In particular, only ZT 0–3 was classified as the sleep state. The results of these analyses confirm that the sleep-awake cycle under the 2 lx condition was similar to the L/D condition, whereas that for the 4 lx condition was not.

The θ-γ CFC has been linked to cognitive processing and was extracted from ECoG obtained over 10 days under various lighting conditions following the entrainment period. As can be seen in Fig. 2f, the θ-γ CFC values were low during daytime when nocturnal rodents are sleeping and high during nighttime when they are awake. These periodic amplitudes were most clearly visible for the L/D condition, while only the periodicity was maintained for the 2 lx condition. However, an apparent daytime–nighttime separation of θ-γ CFC amplitudes was not visually evident under the 4 lx condition.

As can be seen in Fig. 2g, the daily average θ-γ CFC peak value at ZT 12–14 (the two-hour period after waking) was the highest for the L/D condition, which decreased by 34% and 40% for the 2 lx and 4 lx, respectively. The top 20% daily θ-γ CFC values were obtained to evaluate how well high θ–γ CFC levels were preserved over time under each lighting condition (Fig. 2h). On the first day, all three lighting conditions showed comparable values, indicating similar initial levels of high θ–γ CFC. Over the 10-day period, the L/D and 2 lx conditions showed some fluctuations in this metric but overall maintained relatively stable and comparable levels. In contrast, the 4 lx condition exhibited a clear and progressive decline in the top 20% θ-γ CFC values over time. The lowest top 20% θ-γ CFC values observed during the 10-day period for the L/D, 2 lx, and 4 lx conditions were 0.255, 0.281, and − 0.0096, respectively.

To quantitatively assess how well high θ–γ CFC levels were sustained under the different lighting conditions, the distribution of the top 20% θ-γ CFC values over the 10-day period was analyzed using a box plot (Fig. 2i). The L/D condition showed the highest median value with a narrow interquartile range, indicating consistent maintenance of peak θ-γ CFC levels over time. The 2 lx condition exhibited a slightly lower median but retained a relatively compact distribution, suggesting moderate preservation of high θ-γ CFC levels despite nighttime light exposure. In contrast, the 4 lx condition exhibited slightly lower median values and increased variability compared to L/D and 2 lx, showing a broader interquartile range, suggesting a reduced consistency in maintaining a high θ-γ CFC level across the observation period. Statistical comparisons confirmed that there was no significant difference between the θ-γ CFC levels for the L/D and 2 lx conditions, while both had statistically significantly higher levels than those for the 4 lx condition, while both had statistically significantly higher levels than those for the 4 lx condition (Welch one-way ANOVA; *p* = 2.45 × 10^− 3^, η^2^ = 0.463).

We also analyzed whether the awake and sleep states were clearly distinguishable through GMM analysis and *k*-means clustering from a circadian rhythm perspective using the θ-γ CFC values (Fig. 2j). GMM analysis with bimodal Gaussian fitting enabled awake and sleep state classification into two peaks for the two states, the intervals between which were 0.39, 0.29, and 0.22 for the L/D, 2 lx, and 4 lx conditions, respectively. Unlike the NREM index results, the awake and sleep states could clearly be distinguished across all three conditions. The *k*-means clustering results revealed the same awake state at ZT 11–24 across all three conditions, with awake/sleep proportions of 54.2%/45.8%, 53.3%/46.7%, and 53.6%/46.4% for the L/D, 2 lx, and 4 lx conditions, respectively. When considering these results together with those in Fig. 2i, higher θ-γ CFC levels were observed for the 2 lx condition than for the 4 lx condition, even though there was no difference in the timing of the awake state between them.

## Post-disruption recovery with sufficiently low non-visual illuminance

The results confirm that the circadian rhythm was well maintained under the 2 lx condition, not only from a physiological perspective (Fig. 1) but also from a sleep and cognition-associated neural metrics perspective (Fig. 2). Therefore, we investigated whether the 2 lx condition could be used as a meaningful method for correcting and fixing disrupted circadian rhythms.

**Fig. 3:**
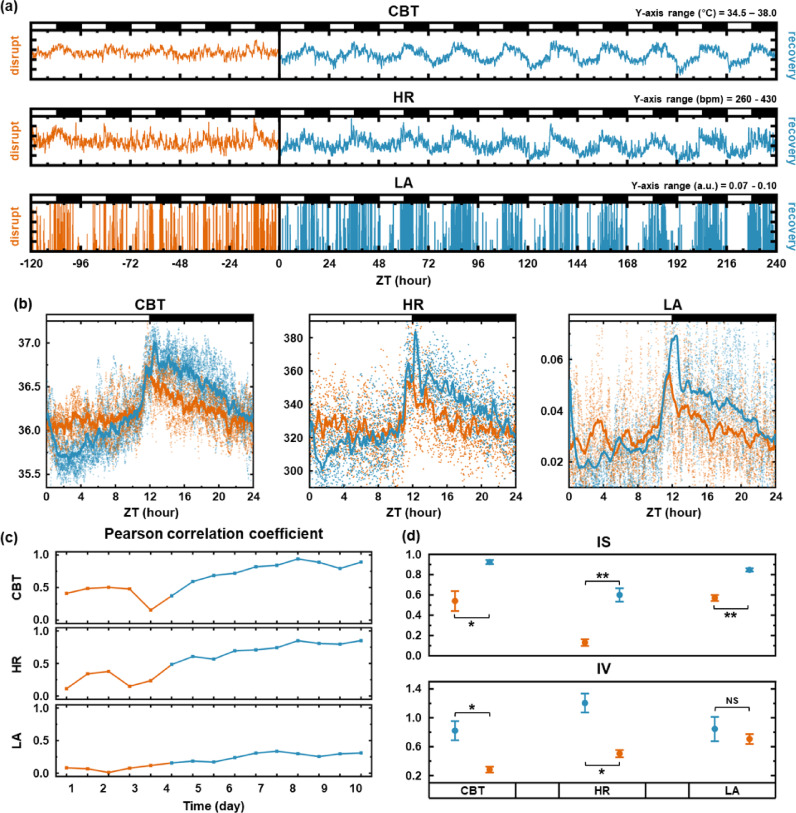
Physiological parameters and circadian rhythm metrics before and after switching from L/L to 2 lx. The circadian disruption phase (5 days) under L/L is shown in orange, while the circadian recovery phase (10 days) under 2 lx is shown in blue. **a** Time-series plots and **b** daily averaged plots for CBT, HR, and LA. **c** Pearson’s correlation coefficient (*r*) values between the daily and L/D reference profile values for CBT, HR, and LA. **d** IS and IV metrics for CBT, HR, and LA. Black/white bars indicate nighttime/daytime, respectively. *n* = 5 (within-animal pre–post). Two-tailed paired t-tests with Holm–Bonferroni correction for multiple comparisons: *p < 0.05, **p < 0.01, ***p < 0.001.

Physiological changes were measured over 10 days after switching to the 2 lx condition at ZT 0 following a circadian-disruption period under constant light (L/L) condition (*n* = 5) (Fig. 3a). Periodicity was not observed in the CBT, HR, and LA plots during the entrainment period. During the subsequent 10-day recovery phase under 2 lx lighting, circadian organization progressively recovered, as indicated by gradual increases in the cyclical amplitude for CBT and HR. In addition, the circadian pattern of LA re-emerged, with reduced activity during daytime and increased activity during nighttime. These changes were more evident in the daily averaged profiles, where daytime values decreased and nighttime values increased after switching to 2 lx lighting; accordingly, the peak-to-peak values for CBT, HR, and LA ​​increasing by 79.2%, 101%, and 60.5%, respectively (Fig. 3b).

The recovery trajectory before and after the switch to 2 lx lighting was further assessed using in terms of linear correlation using Pearson’s *r* (Fig. 3c). During the initial circadian-disrupted state (days − 5 to − 1), the average Pearson’s *r* values for CBT, HR, and LA were 0.408, 0.245, and 0.072, respectively. During the recovery phase (days 6 to 10), these values increased to 0.872, 0.810, and 0.300, representing increases of 114%, 231%, and 319%, respectively. This marked improvement in linear correlation across all biosignals indicates recovery of the circadian temporal patterns following the switch to 2 lx lighting. The improvement in circadian rhythm was also confirmed by IS and IV analysis (Fig. 3d). For all of the biosignals, IS and IV tended to increase and decrease, respectively, after the switch. IS increased from 0.570 to 0.871 for CBT(Holm-adjusted *p* = 0.0480; Cohen’s *d*_z = 2.54), from 0.130 to 0.417 for HR(Holm-adjusted *p* = 0.00872; Cohen’s *d*_z = 8.71), and from 0.570 to 0.817 for LA(Holm-adjusted *p* = 0.00494; Cohen’s *d*_z = 14.21). IV decreased from 0.821 to 0.282 for CBT(Holm-adjusted *p* = 0.0214; Cohen’s *d*_z = 2.27), from 1.204 to 0.503 for HR(Holm-adjusted *p* = 0.0214; Cohen’s *d*_z = 2.08), and from 0.844 to 0.705 for LA(Holm-adjusted *p* = 0.380; Cohen’s *d*_z = 0.44). Except for LA in the IV analysis, most comparisons showed statistically significant changes before versus after the switch, providing evidence of improved circadian rhythms.

**Fig. 4:**
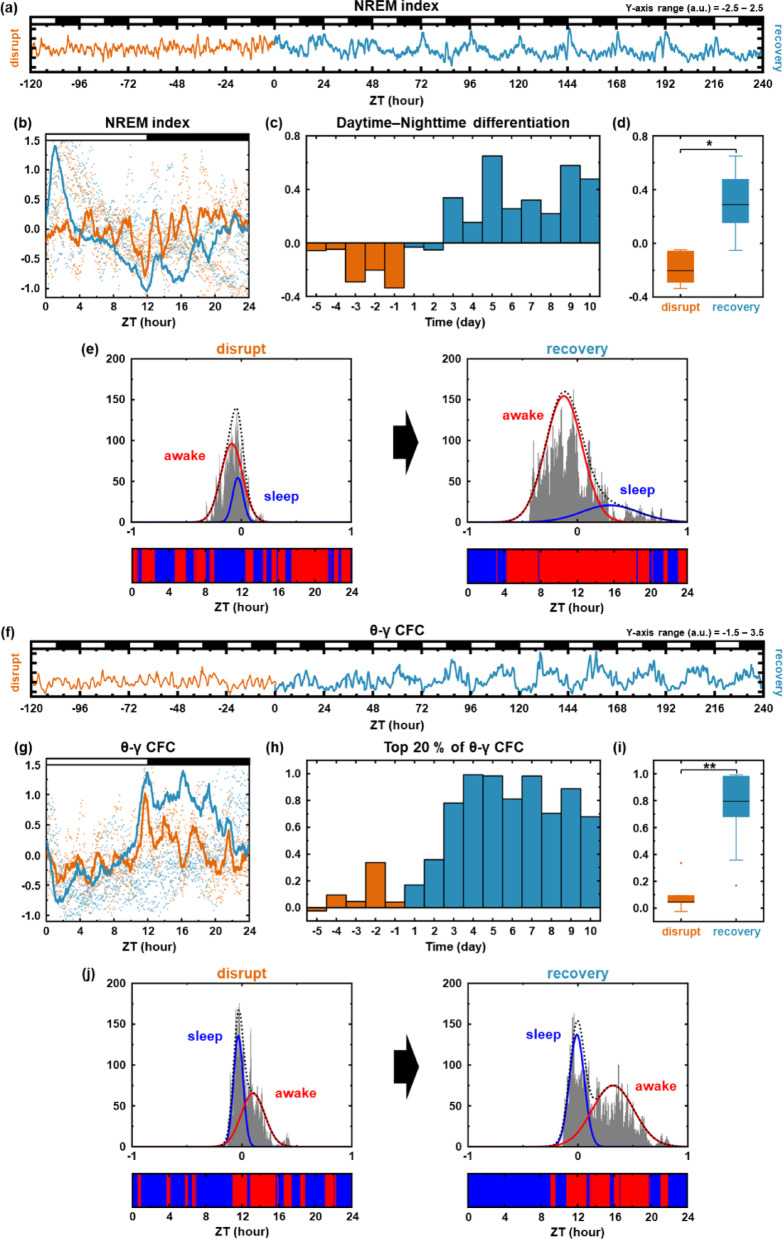
Neurophysiological parameters and circadian rhythm metrics before and after switching from L/L to 2 lx. The circadian disruption phase (5 days) under L/L is shown in orange, while the circadian recovery phase (10 days) under 2 lx is shown in blue. **a** Time-series plots of the NREM index values. **b** Daily averaged NREM index values. **c** Daytime–nighttime differences in NREM index values. **d** A box plot of the daytime–nighttime difference in the NREM index values before and after light therapy. **e** (Top) GMM distribution of NREM index values with bimodal Gaussian fitting to classify the sleep (blue) and awake (red) states, and (bottom) *k-*means clustering results for the NREM index values with two predefined clusters for the sleep and awake states. **f** Time-series plots of the θ-γ CFC values. **g** Daily averaged θ-γ CFC values. **h** Top 20% θ-γ CFC values. **i** A box plot of the top 20% θ-γ CFC values. **j** (Top) GMM distribution of θ-γ CFC values with bimodal Gaussian fitting to classify the sleep (blue) and awake (red) states, and (bottom) *k-*means clustering results of the θ-γ CFC values with two predefined clusters for the sleep and awake states. Black/white bars indicate nighttime/daytime, respectively. *n* = 5 (within-animal pre–post). Two-tailed paired t tests with Holm–Bonferroni correction for multiple comparisons: *p < 0.05, **p < 0.01, ***p < 0.001.

NREM index analysis before and after switching to 2 lx lighting showed a progressive daytime increase beginning around the 4th day of the recovery phase, with peaks at ZT 0–2 (Fig. 4a) and in the daily averages (Fig. 4b).The peak-to-peak value in Fig. 4b increased by 106% after the switch, indicating improved day–night pattern in NREM index relative to the disrupted state. The difference in NREM index values between daytime and nighttime gradually increased from − 0.336 to 0.649 (Fig. 4c), indicating that the circadian rhythm had clearly been recovered. As shown in Fig. 4d, the difference in NREM index values between daytime and nighttime increased after the switch, with a shift in the median from negative to positive values(Holm-adjusted *p* = 0.0354; Cohen’s *d*_z = 1.40).

The GMM analysis was unable to classify the sleep and awake states when the circadian rhythm was disturbed but could do so during the 2 lx recovery phase, with the interval between the two peaks increasing substantially from 0.06 to 0.42 (Fig. 4e). Similarly, *k-*means clustering showed that, during disruption, the awake and sleep states were not synchronized to daytime and nighttime and were not distinguishable from each other, whereas after switching to 2 lx lighting, the two states became clearly separable, indicating improved circadian organization. The awake/sleep proportions were 55.7%/44.3% during disruption, and 71.5%/28.5% during the recovery phase, respectively.

The θ-γ CFC analysis performed to assess cognition-associated neural metrics revealed that the circadian rhythm gradually recovered, peaking at ZT 12–14 from the 3rd day after switching to 2 lx lighting. As can be seen in Fig. 4f, the θ-γ CFC curve during the recovery phase showed a clear increase across ZT 12–20 and a noticeable decrease during ZT 0–4, thus indicating an overall enhancement in nighttime θ-γ CFC levels and suppression during the daytime (Fig. 4g). Changes in the top 20% θ-γ CFC over time showed a clear improvement after switching to 2 lx lighting (Fig. 4h), increasing from − 0.025 during disruption to 0.992 during recovery. As shown in Fig. 4i, the top 20% θ-γ CFC values increased after the switch, with an upward shift in the median and a broader spread(Holm-adjusted *p* = 0.0037; Cohen’s *d*_z = 2.72). In line with the NREM index results, GMM analysis also indicated improved circadian organization after switching to 2 lx lighting, as confirmed by the sleep and awake states being classifiable into two peaks and the interval between them increasing from 0.13 to 0.33 (Fig. 4j). The *k*-means clustering similarly showed that the awake and sleep states became clearly distinguishable after the switch. The awake/sleep proportions were 36.1%/63.9% during disruption and 39.7%/60.3% during the recovery phase, respectively.

## Discussion

To establish a healthy lighting environment, it is important to understand the non-visual effects of light, which are mediated primarily by melanopsin-expressing ipRGC pathways and shaped by integrated retinal inputs, including contributions from rods and cones.^25,26,35^ Although correlated color temperature (CCT) has been widely used as an indicator to intuitively understand the color change of light according to wavelength, it is not suitable for expressing the biological efficacy of light.^36^ Accordingly, the Commission Internationale de l’Eclairage (CIE) recommended melanopic EDI as a predictor of the non-visual effects of light, and it has since been widely adopted to quantify the effects of light exposure on circadian rhythms.^37–41^.

An artificial lighting environment that minimizes the adverse effects of light on circadian rhythms has not yet been designed from a non-visual illuminance perspective and comprehensively evaluated using physiological and ECoG-derived neurophysiological metrics. Ideally, such lighting should not only minimize circadian disturbances but also help restore disturbed circadian rhythms, particularly for shift workers exposed to light at inappropriate times. Accordingly, a healthy nighttime lighting environment requires the minimum necessary VIL with a sufficiently low melanopic EDI. In the present study, we analyzed the effects of two lighting environments illuminated at 2 lx and 4 lx (i.e., the same VIL but different melanopic EDI values) on circadian rhythms.

Under the 2 lx condition, changes in the circadian rhythms of physiological biosignals CBT, HR, and LA were very small compared to the 4 lx condition. In addition, when comparing the biosignal levels obtained under the 2 lx condition with those under the ideal L/D condition, the periodic amplitudes of the biosignals were well maintained, their 24-hour periodicity also remained high, their Pearson’s *r* values were high, and their values for IS and IV (used to evaluate circadian rhythms) were also well maintained. Moreover, ECoG-derived neurophysiological analyses revealed that the periodic amplitudes for the NREM index and θ-γ CFC were well maintained, NREM index values were high during daytime and low during nighttime, the top 20% of the θ-γ CFC values remained high, the sleep and awake states were clearly classifiable, and synchronization between daytime and nighttime was well maintained. In summary, under the 2 lx condition, physical changes were minimal, ECoG-derived neurophysiological metrics remained well organized, and circadian rhythms were not disturbed.

These results are consistent with previous studies that filtering out short-wavelength light below 480 nm during nighttime exposure minimizes disruption of circadian rhythms.^42,43^ The present study extended these findings, demonstrating how changes in melanopic EDI, induced by modulating light intensity within the range of wavelength between 420 and 500 nm, affect circadian rhythms while maintaining constant VIL. In particular, our finding suggest that even the small melanopic EDI change between 2 and 4 lx was associated with substantial differences in circadian rhythms, as confirmed by the observed changes in physiological and ECoG-derived neurophysiological metrics that would have been missed if we had used actograms only.

Our findings indicate that switching to the 2 lx condition after circadian disruption was associated with progressive recovery of circadian rhythms across physiological and neurophysiological endpoints. This was confirmed by improvements in the periodic amplitude and periodicity of the physiological biosignals, NREM index, and θ-γ CFC; Pearson *r* values for comparison with the L/D condition for IS and IV; and daytime and nighttime differences in the NREM index and top 20% of θ-γ values. Moreover, the sleep and awake states could be clearly distinguished. These results suggest that the 2 lx condition may support recovery of disturbed circadian rhythms as assessed by physiological and neurophysiological endpoints. We note that, while the 2 lx condition can still impose a modest perturbation relative to L/D, the comparative findings in Experiment 1 confirm that it supports substantially more organized rhythmicity than the 4 lx condition.

Several limitations should be acknowledged. First, although θ–γ CFC has been linked to cognitive processing in rodents, no learning or behavioral assays were performed in this study; therefore, θ–γ CFC should be interpreted as an indirect ECoG-derived neurophysiological metric reflecting neural state and network coupling rather than a direct measure of cognition. Second, rat mel EDI provides a practical melanopsin-weighted optical metric for comparing spectra, but it does not directly quantify in vivo melanopsin pathway activity, and circadian photoreception reflects integrated retinal signaling and species differences. Accordingly, we report both rat and human mel EDI values for each condition based on the measured spectra (Table S1); the 4 lx condition yielded identical rat and human mel EDI values, whereas the 2 lx condition showed only a small difference, and direct generalization to humans remains limited without further validation. In addition, only male SD rats were used, and sex differences in circadian behavior and light sensitivity/response have been reported.^44,45^ Moreover, as this strain is albino, light sensitivity may differ from that of pigmented species, including humans. Additionally, we did not measure hormonal or molecular circadian markers, so the present conclusions rely on physiological and ECoG-derived endpoints rather than direct biochemical or molecular readouts of circadian entrainment. Finally, sensitivity analyses indicated that the study was adequately powered to detect large effects, whereas smaller biologically meaningful differences may have remained undetected.

In conclusion, the 2 lx condition may support the maintenance and recovery of circadian rhythms as reflected by physiological and neurophysiological endpoints in this rodent model. Together, our findings suggest that fine-tuning the spectral composition of nighttime illumination may help minimize circadian disruption and support recovery after disruption when light exposure is unavoidable. These results provide preliminary evidence to inform circadian-conscious lighting design, though validation in additional species, both sexes, and ultimately in humans will be required.

## Methods

### Animals

All experimental protocols were approved by the Kookmin University Institutional Animal Care and Use Committee (approval number: KMU-2022-03), all experiments were conducted in accordance with ARRIVE guidelines, and all methods were performed in accordance with the relevant guidelines and regulations. Male SD rats (250–350 g, 8 weeks old; Koatech, South Korea) were used. They were housed in individual cages and had access to food and water *ad libitum*.

### Surgery

Rats were anesthetized with 5% isoflurane initially and with 1–2% isoflurane during surgery, with body temperature maintained at 37 °C. An implantable wireless telemetry device (HD-S02, Data Science International, United states) was implanted subcutaneously on the dorsal side, and electrocardiogram (ECG) electrodes were positioned in lead II configuration (Fig. S3a). Screw electrodes were inserted into holes drilled at pre-designated coordinates in the skull (Fig. S3b), and then all incisions were sutured and disinfected.

### Individual light-controllable cage

To individually control the lighting environment for each rat, the cages were constructed from black acrylic to prevent light transmission (inside dimensions: a width of 40 cm, a depth of 50 cm, and a height of 70 cm) (Fig. S2a). A light source consisting of tunable four-package white LEDs^33^ was located on the interior ceiling, and the interior walls were plastered with white sheets to spread the light evenly. A receiver for communication with the telemetry implanted in the rat was located on the bottom, and a transparent rat housing cage was placed on top (Fig. S2b). Sufficient ventilation fans were located on the walls to maintain a constant temperature and humidity within the cage, which was monitored with a thermohygrometer.

### Lighting conditions

Four lighting conditions were designed to operate in the individual light-controllable cages: L, CIL-H for daytime (08:00–20:00, ZT 0–12), and CIL-L 2 lx and 4 lx for nighttime (20:00–08:00, ZT 12–24). The CIL-H condition to imitate sunrise by emphasizing the blue wavelength band has the same VIL as the L condition but with a higher rat mel EDI (216 vs. 161 lx). The two CIL-L conditions that imitate sunset had rat mel EDIs of 2 and 4 lx, with slight differences in the blue wavelength range but with the same VIL and a slight difference in CCT based on 1650 K (Table S1). Spectral irradiance distributions are provided in Fig. S1, and rat mel EDI values were using the Rodent Irradiance Toolbox.^32^.

## Protocols

### Experiment 1 (comparative lighting exposure)

After one week of postoperative recovery, the rats underwent L/D entrainment for two weeks. Afterward, the rats were exposed to the L/D, 2 lx, or 4 lx condition, with the time point of light change coinciding with ZT 0 (L/D, *n* = 11; 2 lx, *n* = 9; 4 lx, *n* = 7).

### Experiment 2 (post-disruption recovery)

After one week of postoperative recovery, the rats were exposed to L/L condition for two weeks as a disruption phase. Afterward, the rats were exposed to the 2 lx condition, with the time point of the light change coinciding with ZT 0 (*n* = 5).

### Biosignal acquisition and analysis

CBT, LA, ECG, and ECoG measurements were obtained via the wireless telemetry HD-S02 devices implanted in the rats. Data were acquired every minute for CBT and LA, and at 500 samples per second for ECG and ECoG. HR was extracted from the ECG using the Pan–Tompkins algorithm. Periodograms were computed using FFT and smoothed using B-splines; prior to FFT-based periodogram estimation, the time series were mean-centered and detrended to reduce slow drifts and non-stationary components. IS and IV were extracted using the formula in ref. 37. The acquired ECoG signals were segmented into 10-s epochs. The minor low-frequency noises and artifacts, including motion artifacts and DC drift, were automatically eliminated using empirical mode decomposition. Specifically, the signals were decomposed into intrinsic mode functions (IMFs) and a residual component, and IMFs with fewer than 10 zero-crossings (corresponding to frequencies below approximately 0.5 Hz), as well as the residual, were removed prior to further analysis. The NREM index values were calculated by applying the formula in ref.^46^, and the θ-γ CFC was extracted using a modulation index-based algorithm^47,48^; the calculated indices were individually normalized using z-score transformation based on the entrainment dataset. GMM analysis and *k*-means clustering were applied to the grand-averaged NREM and θ–γ CFC indices, both assuming two predefined states: sleep and awake. All biosignal analyses were performed using MATLAB.

### Statistical analysis

All values are expressed as the mean ± standard error of the mean. Prior to statistical analyses, the assumptions that the data distribution displayed normality and homoscedasticity were verified using Kolmogorov-Smirnov and Levene tests. For Experiment 1 (between-group comparisons), omnibus effects across lighting conditions were evaluated using Welch one-way ANOVA, followed by two-tailed Welch’s two-sample t-tests for pairwise contrasts, with Holm–Bonferroni correction applied for multiple comparisons. Two-tailed paired t-tests were applied for Experiment 2 (within-animal pre–post comparisons), and Holm–Bonferroni correction was applied when multiple within-animal comparisons were performed. Sensitivity analyses indicated that, at α = 0.05 and 80% power, the minimum detectable effect sizes were approximately Cohen’s d = 1.33, 1.44, and 1.52 for the three pairwise comparisons in Experiment 1 and Cohen’s d_z = 1.68 for the paired comparisons in Experiment 2. Effect sizes are reported alongside *p* values as η^2^ for omnibus effects and Cohen’s *d*_z for within-animal contrasts. Exact *p* values are reported in the Results; for simplicity, *p* values of < 0.05, < 0.01, and < 0.001 are reported as *, **, and ***, respectively. Statistical analyses were conducted using MATLAB with the Statistics and Machine Learning Toolbox.

## Supplementary Information

Below is the link to the electronic supplementary material.

Supplementary Material 1

## Data Availability

The data in this study are available on request from the corresponding author upon reasonable request.
