# Supplementary material for: Melanopic equivalent daylight illuminance of 2 lx maintains and restores physiological and neurophysiological circadian rhythms in rats
Source: Sci Rep. 2026 Apr 25;16:19204. doi: 10.1038/s41598-026-49695-6 (PMC13284233; doi:10.1038/s41598-026-49695-6)
Supplement: Supplementary file 1 — Supplementary Material 1 [file 41598_2026_49695_MOESM1_ESM.docx]

**Supplementary Information**


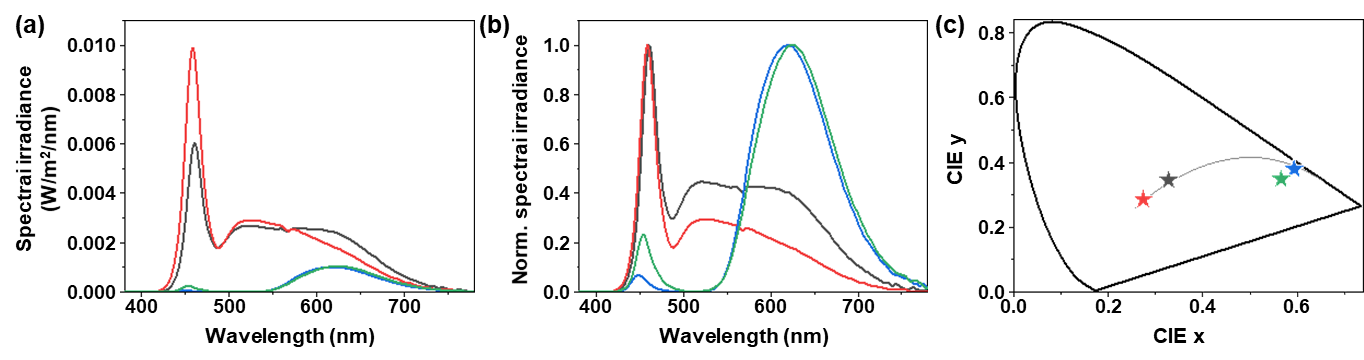


**Fig. S1. Spectral characteristics of the four experimental lighting conditions.** (a) Spectral irradiance, (b) normalized spectral irradiance, and (c) 1931 Commission Internationale de l'Eclairage (CIE) chromaticity coordinates. L, light (black); CIL-H, circadian illuminance high (red); CIL-L, circadian illuminance low with 2 lx or 4 lx rat melanopic equivalent daylight illuminance (rat mel EDI) (blue and green, respectively).

**Table S1. The four experimental lighting conditions.**

|  | VIL (lx) | Rat  mel EDI (lx) | Human  mel EDI (lx) | CCT (K) |
| --- | --- | --- | --- | --- |
| L | 185 | 161 | 175 | 5700 |
| CIL-H | 185 | 216 | 225 | 10000 |
| CIL-L (2 lx) | 30 | 1.6 | 1.8 | 1630 |
| CIL-L (4 lx) | 30 | 3.9 | 3.9 | 1660 |

VIL, visual illuminance; mel EDI, melanopic equivalent daylight illuminance; CCT, correlated color temperature.
Light-emitting diode (LED) peak wavelengths of the tunable four-package white LEDs^33^: Blue 450 nm; Green 520 nm; Amber 590 nm; Red 640 nm.


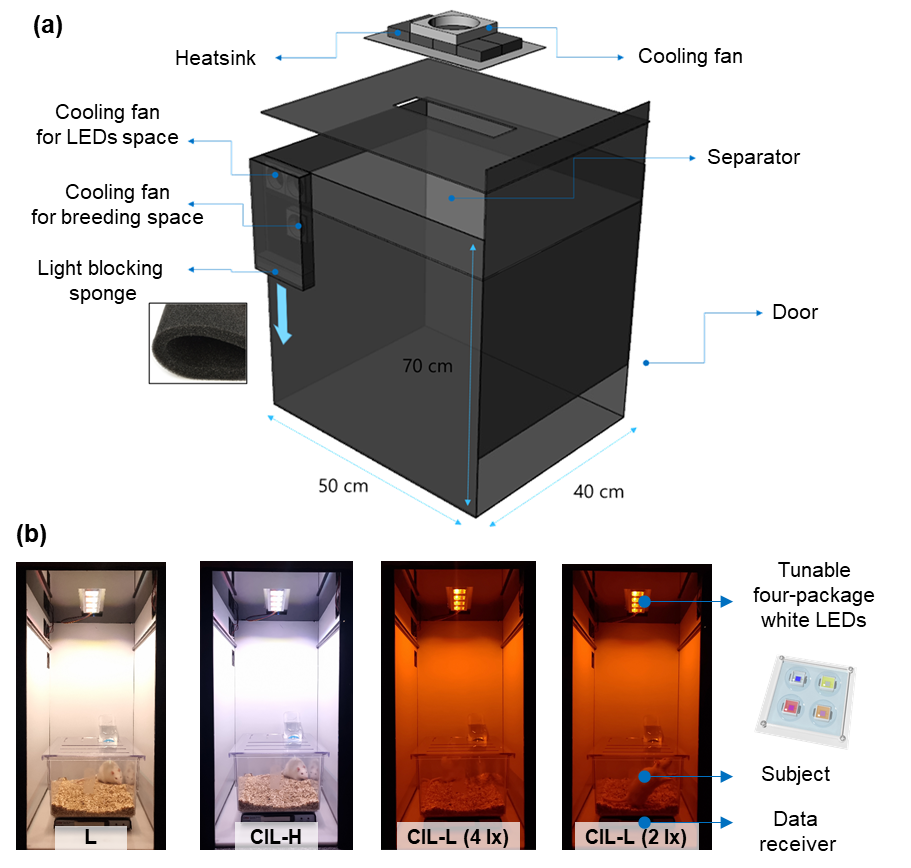


**Fig. S2. Design and setup of the individual light-controllable cage system used for the circadian rhythm experiments.** (a) A schematic diagram of the custom-built light-controllable cage (70 × 50 × 40 cm) featuring an upper-mounted cooling fan and heatsink, internal cooling fans for LEDs and breeding space, light-blocking sponge for isolation, and a removable separator and door for access and maintenance. (b) Photographs of the cage interior under the four experimental lighting conditions (L, CIL-H, and CIL-L with either 4 or 2 lx rat mel EDI). Each cage with a subject implanted with a wireless biosignal acquisition device inside is equipped with tunable four-package white LEDs^33^ and a data receiver for signal collection.


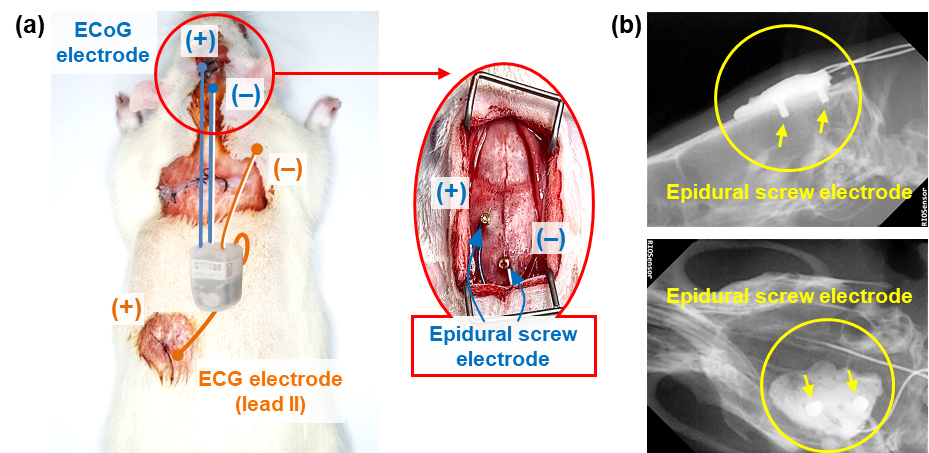


**Fig. S3. Electrode implantation setup for electrocardiogram (ECG) and electrocorticogram (ECoG) monitoring using a wireless telemetry device in rats.** (a) Surgical configuration showing implantation of an implantable telemetry system connected to ECG and ECoG electrodes. The ECG electrodes (orange) were placed in the lead II configuration, while the ECoG electrodes (blue) were placed epidurally via screw electrodes inserted into the skull. A close-up image on the right highlights the epidural screw electrodes placed at pre-designated coordinates on the skull. All electrodes were connected to a subcutaneously implanted wireless telemetry device. (b) X-ray images of the implanted epidural screw electrodes for ECoG recording. The upper image shows a lateral view, and the lower image shows a dorsal (top-down) view, with arrows indicating the positions of the epidural screw electrodes.
